# *In vivo* microdialysis and *in vitro* HPLC analysis of the impact of paeoniflorin on the monoamine levels and their metabolites in the rodent brain

**DOI:** 10.1051/bmdcn/2019090211

**Published:** 2019-05-24

**Authors:** Yuh-Tzy Lin, Wei-Shih Huang, Huei-Yann Tsai, Min-Min Lee, Yuh-Fung Chen

**Affiliations:** 1 Department of Nursing, Jen-Teh Junior College of Medicine, Nursing & Management Miaoli 356 Taiwan; 2 Department of Neurology, China Medical University Taichung 404 Taiwan; 3 Department of Neurology, China Medical University Hospital Taichung 404 Taiwan; 4 Department of Pharmacy, China Medical University Hospital Taichung 404 Taiwan; 5 Department of Food Nutrition and Health Biotechnology, Asia University Taichung 413 Taiwan; 6 Department of Pharmacology, China Medical University Taichung 404 Taiwan

**Keywords:** Paeoniflorin, Monoamines and metabolites, Microdialysis, HPLC analysis, Rodent brain

## Abstract

Background: Paeoniflorin (PF) possesses several effects such as analgesic, the anti-spasmodic effect on smooth muscle. It protects the cardiovascular system and reveals the neuroprotective effect on cerebral ischemia. Monoamine system has been identified to have complex regulatory effects in pain signaling. There are no reports regarding the impact of PF on monoamine levels in the rodent brain by microdialysis. In this study, the effects of PF on monoamines and their metabolites in the rodent brain using *in vivo* microdialysis and *in vitro* high performance liquid chromatography (HPLC) analysis.

Methods: Male S.D. rats were anesthetized, fixed onto the stereotaxic instrument to identify the positions of corpus striatum and cerebral cortex. Drilled a hole in the skull of anesthetic rats and proceeded microdialysis, and gave PF (100 μg, i.c.v.). Collected the dialysate and the concentration of monoamines and their metabolites in dialysate and analyzed with HPLC-ECD. Male ICR mice were administered with PF (96 μg, i.c.v.) and with Ringer solution as a control. After 20 mins of administration, the mice were cut off the brain immediately and separated into eight regions according to the method of Glowinski. Added extraction solution to each region, homogenized and extracted for further procedure. The extract was centrifuged, sucked the transparent layer and centrifuged once more. The transparent layer was filtered with a 0.22 μm nylon filter and analyzed with HPLC-ECD (electrochemical detection).

Results: PF increased the content of DOPAC and NE in the cortex, and increased the content of NE and decreased the content of 5-HT in the medulla of the homogenized mice brain tissue. By microdialysis, PF increased the content of DOPAC and 5-HIAA in anesthetic rat cortex and expanded the content of DOPAC, HVA, and 5-HIAA in anesthetic rat striatum.

Conclusions: It reveals that PF could activate the release of monoamines and increase their metabolites in the rodent brain.

## Introduction

The monoamines have complex modulatory roles in pain signaling, and descending controls can enhance or reduce the transmitted pain signal [1]. The monoamines (norepinephrine and serotonin) are the neurotransmitters majorly implicated in the descending modulatory pathway [2].

Paeony roots (*Paeonia lactiflora* PALL., Ranunculaceae) exhibited anticoagulative activity [3], mild sedative, a potent analgesic, antispasmodic, and neuroprotective effects [4-6]. The analgesic, antispasmodic and neuroprotective properties of paeony roots were *via* its active component paeoniflorin [7]. Paeoniflorin had shown the antinociceptive effects on the writhing response test and the formalin test in mice in our previous studies [8-10]. The antinociception of paeoniflorin on the formalin test in mice was mediated by the activation of κ-opioid receptor [9] and modulation of NMDA receptors [10].

Opioid drugs produced their actions by interacting with three major types of opioid receptors, namely μ-, δ- and κ-opioid receptors, in the analgesic system. In a previous study, the antinociceptive effect mediated by κ-opioid receptor related to the serotonergic neurons [11]. The release of neurotransmitters in the nervous endings might need the influx of Ca^2+^ *via* the calcium channel [12, 13]. The L-type voltage-sensitive calcium channel involves in noradrenaline release in the rat brain [14]. From previous studies, paeoniflorin inhibited calcium influx and blocked intracellular calcium movement [15-20]. The antinociceptive responses of L-arginine were decreased by the L-type calcium channel antagonists, diltiazem or verapamil [21]. It indicated that there were interactions between neurotransmitter release and intracellular calcium in the regulation of nociception.

*In vivo* microdialysis and *in vitro* HPLC analysis of the impact of paeoniflorin on the monoamine levels and their metabolites in the rodent brain were performed in the present study.

## Materials and methods

### Preparation of microdialysis probe before experiments

Use a new microdialysis CMA/12 probe (polycarbonate membrane, Carnegie Medicin; the membrane’s diameter = 0.5 mm, the activity length = 2 or 3 mm) in this study. The new probe had to soak with ethanol for 3-5 mins to remove the protective membrane of glycerol. After this procedure, put the probe into the deionized water and perfused with de-ionized water in 10 μl/min of perfusion rate for 20 mins and with Ringer solution in 10 μl/min for 20 mins, then in 1.2 μl/min for 2 hrs to overnight. It had to perfuse with Ringer solution in 1.2 μl/min for an overnight for the used probe before experimenting.

### Recovery rate of the microdialysis probe

Put the microdialysis probe into the standard solution contained monoamines (NE, EPI, DA, 5-HT) and their metabolites (HVA, DOPAC, 5-HIAA) and connected to the microinjection pump (CMA/100, Carnegie Medicin) and the micro-fraction collector (CMA/140, Carnegie Medicin) to proceed the recovery rate test *in vitro*. The collection vial contained 5 μl ascorbic acid (10^-7^M) to prevent the oxidation of monoamines and their metabolites. Then perfused the probe with Ringer solution in 1.2 μl/min, collected a vial every 20 mins. The 3^rd^~5^th^ collection vials were used to calculate the recovery rate. Tested the recovery rate before and after the experiments called recovery and post-recovery, in order to calibrate the concentrations of monoamines and their metabolites with microdialysis probe in rat’s brain.

### Ethics statement

The IACUC (Institutional Animal Care and Use Committee) of China Medical University approved the experimental protocol, and the permit number was 2018-250.

### Microdialysis with anesthetic rats

**Fig. 1:**
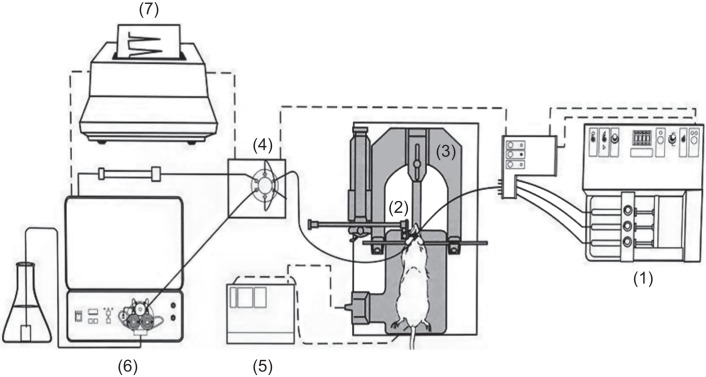
Microdialysis setup for the anesthetized rat. (1) Microdialysis syringe pump, (2) Microdialysis probe placed into the brain, (3) Stereotaxic instrument, (4) Fraction collector, (5) Temperature controller, (6) High-performance liquid chromatography/electrochemical detector (HPLC/ECD), (7) Chromatography chart. (This figure is a slightly modified adaptation of the picture of CMA product catalog with permission of the CMA representative, Anatech Co., LTD., in Taipei).

Male S.D. rats weighing 250-300 g, anesthetized with urethane (1.5 mg/kg, i.p.), then fixed onto the stereotaxic instrument to identify the positions of corpus striatum (A/P: +0.2 mm, M/L: ± 3.0 mm, D/V: -7.5 mm) and cerebral cortex (A/P: 0 mm, M/L: ± 5.5 mm, D/V: -3.0 mm) by the basal line of bregma and dura [22], the anesthetic rats were warmed by temperature controller (CMA/150, Carnegie Medicin). Drilled a hole on the skull of rats in order to correctly insert the microdialysis probe into the striatum or the cortex. Proceeding microdialysis with Ringer solution by microinjection pump in perfusion rate of 1.2 *ml*/min for 1 hr to equilibrium, the rats were given paeoniflorin 100 μg (i.c.v.). We collected the dialysate every 20 mins with collection vial contained 5 μl 10-7M ascorbic acid as an antioxidant. Analyzed monoamines and their metabolites concentration in dialysate with HPLC (PM 80 pump), and ECD (BAS LC-4C, BioAnalytical Systems, West Lafayette INC U.S.A.) used to detect the concentration of monoamines and their metabolites (illustrated with Fig. 1). After the experiments finished, removed the probes, and sacrificed the rats to cut off the brain and fixed with the saline containing 10% formalin and 30% sugar, then identified whether the inserted positions were correct by frozen section.

### The content of monoamines and their metabolites in mice

Male ICR strain mice weighing 28-35 g was administrated with paeoniflorin (96 μg, i.c.v.) and with Ringer solution as a control. After 20 mins of administrating paeoniflorin or Ringer solution, cut off the mice brain immediately and put the brain into the ice. The brain was separated to the following regions of cortex, corpus striatum, medulla oblongata, and spinal cord according to the method of Glowinski [23]. Added 2 *ml* extraction solution (10^-7^ M ascorbic acid, 1.5 mg pargyline in 0.1N HCl /100 *ml*) to each region and homogenized with a homogenizer (GT-R Eyela^®^); extracted with 3 *ml* extraction solution for twice. Centrifuged the extract with 10000 × g for 20 mins in 4°C, then sucked the transparent layer and centrifuged once more. Finally, filtered the transparent layer with a 0.22 μm nylon filter and analyzed with HPLC-ECD to detect the content of monoamines and their metabolites.

### HPLC analysis

The analytic HPLC column was semimicrobore inertsil (ODS-2, 5 μm, 1.0 × 150 mm). The compositions of mobile phase were 950 *ml* MCA (monochloroacetic acid) buffer (each liter contain 9.45 g/l monochloroacetic acid, 186 mg/l Na2-EDTA, 160 mg/l sodium 1-octane-sulfonate, pH 2.9) combined 50 *ml* acetonitrile. The mobile phase was filtrated by 0.22 μm filter and degassed by high pure nitrogen for 20 mins before use. The flow rate of HPLC was 50 μl/min. The potentials of ECD on working oxidation and reduction electrodes were 0.75 V, and 0.05 V, the range and filter of both electrodes were 10 nA and 0.1 Hz, respectively. The HPLC, connected with an auto-sampling system (refrigerated microsampler, CMA-200, Carnegie Medicin, CMA/microdialysis AB), and the data analyzed with the software of SIC chromatography recorder.

### Preparation of standard curve of monoamines and their metabolites

**Fig. 2:**
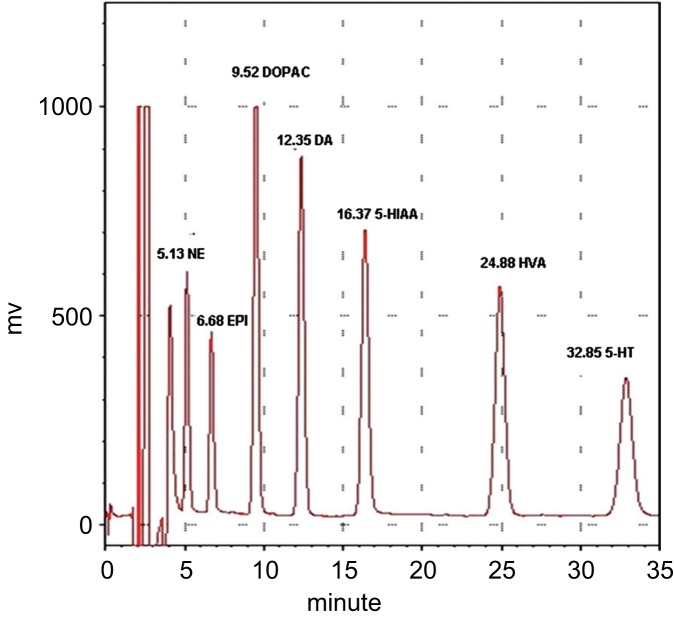
The analysis of a mixed standard solution of monoamines and their metabolites with chromatogram of high-performance liquid chromatography and an electrochemical detector (HPLC-ECD), and the retention time of each peak. NE: norepinephrine; EPI: epinephrine; DOPAC: 3.4-dihydroxyphenylacetic acid; DA: dopamine; 5-HIAA: 5-hydroxyindoleacetic acid; HVA: homovanillic acid; 5-HT: serotonin.

**Fig. 3:**
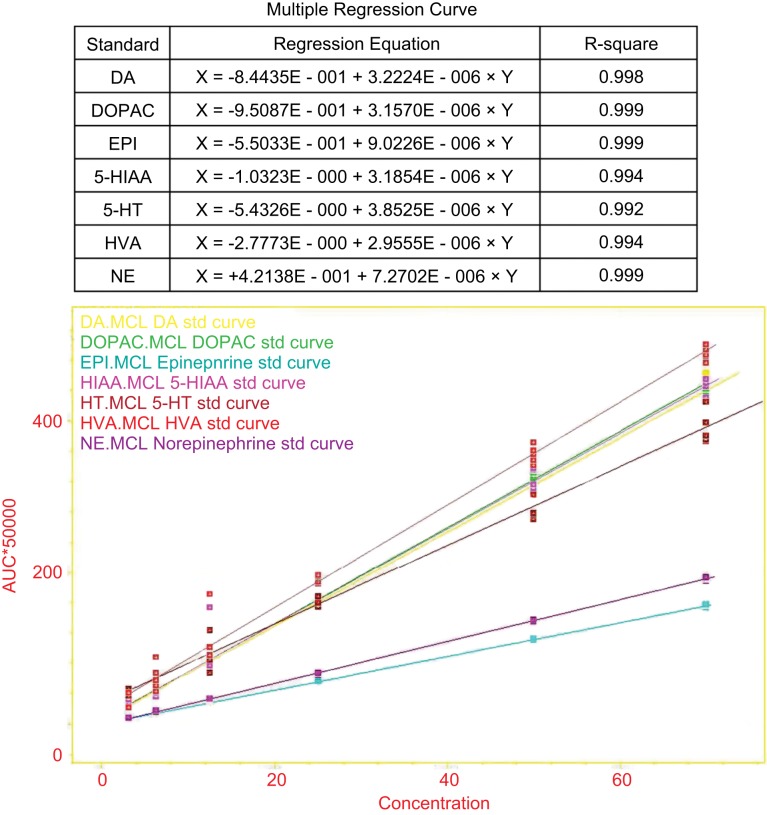
Multiple Regression curve. (Upper): Analytical data for the seven standards in the HPLC-ECD. X and y represent the concentration of the standard (μmol/L) and the peak current (μA), respectively. (Lower): Concentrations and AUC of the seven standards.

0.1 M HClO4 solution was prepared to dissolve the standard monoamines (NE, EPI, DA, 5-HT) and their metabolites (HVA, DOPAC, 5-HIAA). Weighing 10 mg of each standard agent, and together dissolved with 0.1 M HClO4, then added adequate deionized water to 1000 *ml* (this called stock standard solution, 100 ng/μl). The 100 ng/μl stock standard solution was diluted with 0.1 M HClO_4_ to become 100 pg/μl, 75 pg/μl, 50 pg/μl, 25 pg/μl, 12.5 pg/μl, 6.25 pg/μl, and 3.125 pg/μl, and quantitatively analyzed with HPLC and SIC chromatography system to make a standard evaluate curve (illustrated with Fig. 2; Fig. 3).

### Statistical analysis

The results were expressed as mean ± S.E. The differences between mean values were compared using one-way ANOVA (post hoc test with Duncan’s test) or the Student *t*-test and were considered statistically significant when *P* < 0.05.

## Results

### Effect of paeoniflorin on the concentration of monoamines and their metabolites in anesthetic rats with microdialysis

The basal level of monoamines of EPI (epinephrine), NE (norepinephrine) and DA (dopamine) at the perfusion of 60 mins was 1.56 ± 0.71, 8.41 ± 1.25 and 0.81 ± 0.22 pg/μl in the cerebral cortex of rats, and was 3.30 ± 1.18, 17.27 ± 4.42 and 0.16 ± 0.10 pg/μl in the corpus striatum of rats, but 5-HT (serotonin) neither in the cerebral cortex nor in the corpus striatum was detectable in rats. The basal level of monoamines’ metabolites of HVA (homovanillic acid), DOPAC (dihydroxyphenylacetic acid) and 5-HIAA (5-hydroxyindole acetic acid) was 50.44 ± 9.58, 54.05 ± 8.89 and 69.07 ± 8.53 pg/μl in the cerebral cortex, and was 53.74 ± 10.93, 61.83 ± 8.65 and 40.87 ± 5.51 pg/μl in the corpus striatum at the perfusion of 60 mins.

**Fig. 4:**
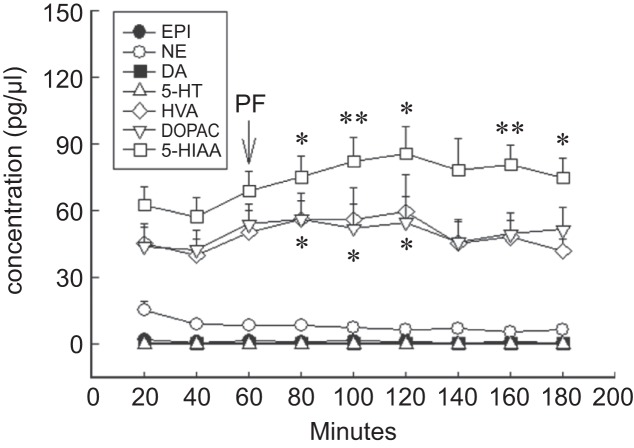
Effect of paeoniflorin (PF) on the content of monoamines and their metabolites with microdialysis of anesthetic rats in the cerebral cortex. Data are shown as mean ± S.E. (n = 8); * *P* < 0.05, ** *P* < 0.01 compared with control.

Paeoniflorin (100 μg, i.c.v.) had no effects on the concentration of monoamines, EPI, NE, DA and 5-HT, and on HVA, metabolite of DA, at the perfusion of 20~120 mins but increased the concentration of DOPAC, metabolites of DA, at the perfusion of 20 mins (56.31 ± 8.43 pg/μl, *P* < 0.05), 40 mins (52.20 ± 11.03 pg/μl, *P* < 0.05) and 60 mins (54.87 ± 11.55 pg/μl, *P* < 0.05), and the concentration of 5-HIAA, metabolites of 5-HT, at the perfusion of 20 mins (75.27 ± 9.50 pg/μl, *P* < 0.05), 40 mins (82.35 ± 10.71 pg/μl, *P* < 0.01), 60 mins (85.81 ± 12.29 pg/μl, *P* < 0.05), 100 mins (80.82 ± 8.70 pg/μl, *P* < 0.01) and 120 mins (74.95 ± 8.99 pg/μl, *P* < 0.05) in the rats’ cerebral cortex dialysate (Fig. 4; Table 1.).

**Table 1:** The effects of paeoniflorin on the concentration of monoamines and their metabolites in the cerebral cortex of anesthetic rats with microdialysis.

pg/*ml*	control			i.c.v. with paeoniflorin (100 mg)					
	20 min	40 min	60 min	20 min	40 min	60 min	80 min	100 min	120 min
EPI	2.25	0.93	1.56	1.03	1.58	1.46	0.85	1.06	0.77
	±0.99	±0.71	±0.71	±0.60	±0.81	±0.78	±0.29	±0.40	±0.40
NE	15.30	9.00	8.41	8.67	7.62	6.66	6.94	5.46	6.75
	±3.98	±1.01	±1.25	±1.89	±1.54	±1.03	±1.00	±0.79	±1.74
DA	0.71	0.58	0.81	0.73	0.55	0.81	0.65	0.55	0.44
	±0.19	±0.46	±0.22	±0.23	±0.23	±0.31	±0.28	±0.28	±0.22
HVA	45.35	40.24	50.44	56.10	56.35	59.66	45.59	48.20	41.93
	±7.67	±7.41	±9.58	±12.02	±13.98	±16.68	±10.62	±7.56	±5.41
DOPAC	44.18	42.52	54.05	56.31*	52.20*	54.87*	46.06	49.73	51.61
	±10.21	±8.65	±8.89	±8.43	±11.03	±11.55	±9.38	±9.65	±10.05
5-HIAA	62.64	57.40	69.07	75.27*	82.35**	85.81*	78.39	80.82**	74.95*
	±8.29	±8.78	±8.53	±9.50	±10.71	±12.29	±14.22	±8.70	±8.99

The concentration of 5-HT was not detectable.

Data are shown as mean ± S.E. (n = 8); * *P* < 0.05, ** *P* < 0.01 compared with control.

**Fig. 5:**
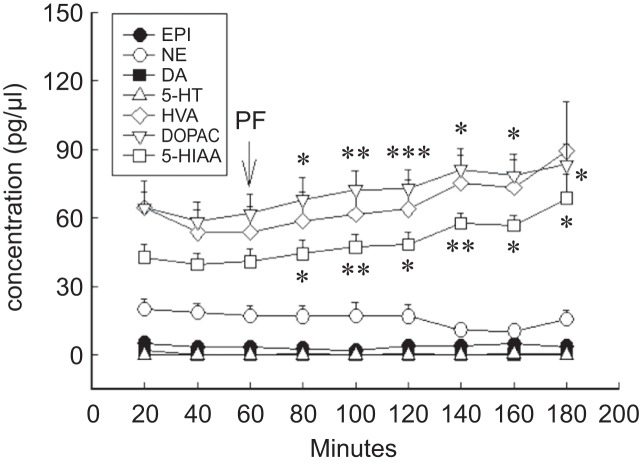
Effect of paeoniflorin (PF) on the content of monoamines and their metabolites with microdialysis of anesthetic rats in the corpus striatum. Data are shown as mean ± S.E. (n = 8); * *P* < 0.05, ** *P* < 0.01, *** *P* < 0.001

In the dialysate of rats’ corpus striatum, paeoniflorin (100 μg, i.c.v.) could not influence the concentration of monoamines, EPI, NE, DA and 5-HT, at the perfusion of 20~120 mins but increase the concentration of HVA at the perfusion of 40 mins (61.57 ± 12.36 pg/μl, *P* < 0.05), 60 mins (63.74 ± 12.87 pg/μl, *P* < 0.05) and 80 mins (75.14 ± 15.27 pg/μl, *P* < 0.05), and the concentration of DOPAC at the perfusion of 20~120 mins (68.03 ± 9.38 pg/μl, *P* < 0.05; 72.29 ± 8.22 pg/μl, *P* < 0.01; 72.90 ± 8.31 pg/μl, *P* < 0.001; 81.02 ± 6.13 pg/μl, *P* < 0.05; 78.51 ± 7.05 pg/μl, *P* < 0.05; 83.22 ± 9.12 pg/μl, *P* < 0.05) and the concentration of 5-HIAA at the perfusion of 20~120 mins (44.23 ± 6.18 pg/μl, *P* < 0.05; 47.28 ± 5.42 pg/μl, *P* < 0.01; 48.28 ± 5.44 pg/μl, *P* < 0.05; 57.51 ± 4.57 pg/μl, *P* < 0.01; 56.67 ± 4.31 pg/μl, *P* < 0.05; 68.49 ± 10.58 pg/ μl, *P* < 0.05) (Fig. 5.; Table 2.).

**Table 2:** The effects of paeoniflorin on the concentration of monoamines and their metabolites in the corpus striatum of anesthetic rats with microdialysis.

pg/*ml*	control			i.c.v. with paeoniflorin (100 mg)					
	20 min	40 min	60 min	20 min	40 min	60 min	80 min	100 min	120 min
EPI	5.17	3.53	3.30	2.69	1.93	3.78	3.86	4.77	3.66
	±1.76	±1.25	±1.18	±0.96	±0.59	±1.06	±1.84	±1.69	±1.94
NE	20.13	18.43	17.27	16.87	17.22	16.87	10.98	10.50	15.71
	±4.41	±4.16	±4.42	±4.60	±5.79	±5.08	±1.57	±1.82	±3.81
DA	1.87	0.16	0.16	0.32	0.27	0.43	0.10	0.17	0.56
	±1.08	±0.11	±0.10	±0.20	±0.18	±0.22	±0.10	±0.11	±0.26
HVA	64.38	53.49	53.74	58.60	61.57*	63.74*	75.14*	73.11	89.18
	±11.85	±10.11	±10.93	±12.75	±12.36	±12.87	±15.27	±14.80	±21.37
DOPAC	64.37	58.39	61.83	68.03*	72.29**	72.90***	81.02*	78.51*	83.22*
	±6.87	±8.60	±8.65	±9.38	±8.22	±8.31	±6.13	±7.05	±9.12
5-HIAA	42.57	39.49	40.87	44.23*	47.28**	48.28*	57.51**	56.67*	68.49*
	±5.53	±5.18	±5.51	±6.18	±5.42	±5.44	±4.57	±4.31	±10.58

The concentration of 5-HT was not detectable.

Data are shown as mean ± S.E. (n = 7); * *P* < 0.05, ** *P* < 0.01, *** *P* < 0.001 compared with control.

### Effect of paeoniflorin on the content of monoamines and their metabolites in the mice brain

Paeoniflorin (96 μg, i.c.v.) could increase the content of NE (35.08 ± 5.53, *P* < 0.05) and DOPAC (147.50 ± 31.37, *P* < 0.05) in the cortex and of NE (117.36 ± 41.71, *P* < 0.05) in the medulla, but decrease the content of 5-HT (0.00 ± 0.00, *P* < 0.001) in the medulla of mice. Paeoniflorin had no effect on the striatum and spinal cord in mice (Table 3). Serotonin was not detectable in the spinal cord of mice.

**Table 3:** Effects of paeoniflorin (PF, 96 mg, i.c.v.) on the content of monoamines and their metabolites of the brain in mice.

ng/g tissue	Cortex		Striatum		Medulla		Spinal cord	

	control	PF	control	PF	control	PF	control	PF
EPI	19.37	70.11	313.69	204.14	258.84	206.65	115.94	86.07
	±7.45	±24.59	±106.31	±46.31	±74.32	±52.40	±31.63	±13.12
NE	10.98	35.08*	84.62	196.02	44.13	117.36*	19.08	62.24
	±6.69	±3.53	±29.43	±46.06	±28.04	±41.71	±12.31	±39.77
DA	179.07	208.98	998.90	925.11	303.72	365.78	251.01	205.94
	±28.87	±32.40	±364.74	±170.53	±29.29	±56.70	±57.93	±60.21
5-HT	135.90	121.25	54.56	145.12	10.70	0.00***	--	--
	±26.55	±60.79	±43.52	±56.85	±2.69	±0.00		
HVA	35.93	37.40	380.02	279.87	18.96	44.75	28.80	27.37
	±5.79	±5.52	±80.74	±72.70	±1.00	±27.82	±25.24	±17.66
DOPAC	61.00	147.50*	1627.05	1123.21	47.56	64.28	26.64	38.65
	±8.52	±31.37	±231.95	±153.50	±7.76	±7.60	±10.92	±7.46
5-HIAA	34.68	22.09	88.81	105.23	96.66	122.16	109.47	61.10
	±6.57	±10.23	±35.05	±36.03	±24.86	±11.26	±52.68	±14.34

Data are shown as mean ± S.E. (n = 8); * *P* < 0.05, *** *P* < 0.001 compared with control.

## Discussion

Descending pathways originate in midbrain and brainstem regions, and project to the dorsal horn of the spinal cord [1]. The participation of norepinephrine and serotonin in spinal descending inhibition is well known, and a critical role for dopamine in descending inhibition has also been demonstrated [33]. Dopamine was metabolized to HVA and DOPAC by MAO (monoamine oxidase) and COMT (catechol-O-methyl transferase), and serotonin was metabolized to 5-HIAA by MAO. In our experiments, the results showed paeoniflorin (100 μg, i.c.v.) increased the concentration of DOPAC and 5-HIAA, the metabolites of dopamine and serotonin, in the cerebral cortex of anesthetic rats with microdialysis *in vivo*. It indicated that paeoniflorin could increase the turnover rate of dopamine and serotonin in the cerebral cortex of anesthetic rats. In the corpus striatum, paeoniflorin (100 μg, i.c.v.) increased the content of HVA, DOPAC, and 5-HIAA, in anesthetic rats with microdialysis. It indicated that paeoniflorin could also increase the turn over rate of dopamine and serotonin in the corpus striatum of anesthetic rats. Paeoniflorin (96 μg, i.c.v.) could increase the content of NE, and DOPAC in the cortex of mice. In the medulla, paeoniflorin increased the content of NE, but decreased the content of 5-HT of mice (our unpublished data). From the above results, the effects of paeoniflorin in the central nervous system might be related to the catecholaminergic and serotonergic system.

In analgesic systems, the monoamines play an essential role as well as the opioid system. The antinociception of serotonin was related to the descending pathway from the nucleus raphe magnus of medulla [24, 25]. A 5-HT receptor agonist, m-CPP, could attenuate the acute and chronic nociceptive response *via* activation 5-HT1 and 5-HT2 receptors [26]. 5-HT3 receptor antagonists, granisetron and ondansetron, could diminish the nociception of late phase on the formalin test, it revealed that activating 5-HT3 receptor might induce nociceptive response [27]. In our previous studies, we showed the antinociceptive effects of paeoniflorin on writhing response test and formalin test in mice [8, 9], and the analgesic effect of 100 μg paeoniflorin by i.c.v. was equal to the analgesic effect of 150 mg/kg aspirin by intraperitoneal injection. The central antinociceptive effects of paeoniflorin might be mediated by the activation of κ-opioid receptor on formalin test [9]. Recent studies have suggested that 5-HT3 receptor might activate the 5-HT gated cation channel [28]. The antinociception mediated by κ-opioid receptor was related to serotonergic neurons [11]. We considered that it might have some relation between the serotonergic neurons and intracellular calcium.

In the present study, paeoniflorin decreased the content of serotonin in the medulla of mice; it might relate to the increase of 5-HT turnover rate; in the cortex of mice, paeoniflorin increased the content of norepinephrine. In the cortex of rats, L-type voltage-sensitive calcium channel was involved in the noradrenaline release [14]. In radial maze performance of rats, α- and β-adrenergic systems were involved in the effects of paeoniflorin antagonizing the scopolamine-induced deficit [29-32]. We have known that neurotransmitters release in the nervous endings required extracellular calcium influx *via* calcium channel [12, 13]. In previous studies, paeoniflorin inhibited calcium influx and blocked intracellular calcium movement [15-20]. We would suggest that the effects of paeoniflorin on the central nervous system might mediate by the inhibition of intracellular calcium flux. Furthermore, previous reports indicate that serotoninnorepinephrine reuptake inhibitor reveals analgesic effect [34-36]. In the microdialysis experiments, paeoniflorin increased the metabolites of serotonin and dopamine, meaning paeoniflorin could increase the turnover rate of serotonin and dopamine. Paeoniflorin does not change the levels of monoamine but increase the metabolites concentration. It reveals that paeoniflorin might inhibit the reuptake of norepinephrine and serotonin, and increases the turnover of monoamine. However, further evaluation is required to have direct shreds of evidence.
